# Analgesic Efficacy and Safety of Intrathecal Morphine in Surgical Patients: A Narrative Review

**DOI:** 10.7759/cureus.113648

**Published:** 2026-07-30

**Authors:** Chloe P Veilleux, Alison R Omsberg, Janelle M Richard, Aurora N Quaye

**Affiliations:** 1 Anesthesiology, Tufts University School of Medicine, Boston, USA; 2 Anesthesiology and Perioperative Medicine, Maine Medical Center, Portland, USA

**Keywords:** intrathecal morphine, narrative review, opioid analgesics, perioperative care, post-operative pain

## Abstract

It is well established that intrathecal morphine (ITM) provides prolonged postoperative analgesia and reduces systemic opioid requirements across a wide range of surgical procedures. Although extensive evidence supports the efficacy and safety of ITM, concern for delayed respiratory depression persists. Additionally, there is no standard approach for ITM dosing or postprocedural monitoring practices across institutions or medical societies, leading to wide variability that further contributes to inconsistent adoption.

In this narrative review, we synthesize contemporary evidence on the pharmacology, analgesic efficacy, and risk profile of ITM and evaluate how current evidence aligns with existing postprocedural monitoring recommendations. A structured literature review identified clinical trials, observational studies, systematic reviews, and practice guidelines published within the past 20 years that evaluated perioperative ITM use.

Across surgical populations, ITM is associated with reductions in postoperative pain scores and systemic opioid consumption at contemporary doses. At these doses, the incidence of respiratory depression was low and comparable to that observed with intravenous opioids. Reported respiratory events were strongly associated with patient-specific risk factors. Overall, current evidence supports the use of ITM as an effective analgesic modality with an acceptable safety profile. These findings support a risk-stratified approach to postprocedural monitoring that accounts for patient comorbidities and the ITM dose administered.

## Introduction and background

Acute postoperative pain is a common clinical challenge, with a substantial proportion of patients experiencing inadequate pain control following surgery [1]. Intravenous (IV) opioids are the most frequently prescribed treatment for postoperative pain, though they often provide incomplete analgesia when used alone and are associated with dose-dependent adverse effects [2]. Neuraxial opioid administration into the epidural or subarachnoid space offers distinct benefits, including superior pain control, reduced systemic opioid exposure, and more rapid recovery in the post-anesthesia care unit when compared with IV administration [2,3].

Intrathecal morphine (ITM) for pain management was first described by Wang et al. in 1979 [4]. In this study, ITM administered to patients with intractable cancer pain produced sustained pain relief without side effects. Use of ITM in obstetrics and the perioperative period began emerging in the 1980s [5,6]. It has since been described in practice guidelines and enhanced recovery after surgery pathways in several surgical specialties [7].

Administration of ITM involves injecting morphine into the cerebrospinal fluid (CSF) of the subarachnoid space, where it acts on neuraxial mu opioid receptors [8]. Single-injection (ITM) is the most widely used neuraxial technique because it provides prolonged analgesia when compared with other intrathecal opioids and offers practical advantages over continuous epidural analgesia or regional nerve blocks [7,9,10]. ITM is simpler to administer, avoids the need for catheter management, and is associated with fewer procedural complications and lower technical failure rates [7,10-12]. Use of ITM is most established in obstetrics; it is also routinely used in cardiothoracic, abdominal, spinal, urological, and orthopedic procedures [7].

Despite its benefits, ITM use remains controversial because of concerns about side effects - the most prominent of these is the risk of delayed respiratory depression [13,14]. Morphine is known to be a hydrophilic drug that remains at sustained high concentrations in the CSF, allowing for cephalad spread that increases the risk of slow penetration into the brainstem and resultant delayed respiratory depression [15]. A unifying definition of respiratory depression does not currently exist and was described by the American Society of Anesthesiologists in 2016 to encompass parameters including bradypnea, hypoxemia, hypercarbia/hypercapnia, and other clinical signs indicative of decreased respiratory function [16].

Early literature on ITM described a high risk of delayed respiratory depression [17]. However, these findings were reported in the context of routine use of ITM in doses of 1000 µg-4000 µg. According to a 2025 meta-analysis, doses in contemporary practice rarely exceed 500 µg, at which the rate of respiratory depression is thought to be equivalent to that observed with systemic opioids [18]. Evidence suggests that the risk for delayed respiratory depression is dose-dependent, though patients with baseline respiratory dysfunction, oxygen dependence, obstructive sleep apnea (OSA), severe obesity, and advanced age may warrant increased monitoring [19].

Nevertheless, protocols for postprocedural management of patients receiving ITM remain conservative and may even be prohibitive to its use in resource-constrained settings [7,20]. Conversely, some studies report that ITM use may reduce overall monitoring needs compared with high-dose systemic opioids [21,22]. These examples illustrate an underlying misalignment in institutional approaches to neuraxial opioid use and the need for a comprehensive evaluation of postprocedural management recommendations to optimize patient safety while promoting efficient resource utilization.

The purpose of this narrative review is to evaluate the perioperative use of ITM, including its pharmacologic properties, analgesic efficacy, safety profile, and implications for patient monitoring. Additionally, we will examine existing postprocedural monitoring recommendations and evaluate how current evidence aligns with existing practice guidelines.

## Review

Methods

A literature search was performed on PubMed on June 29, 2025, to identify reports on the clinical use of ITM in surgical settings. Search terms included the following: (("intrathecal morphine") OR ("intrathecal opioids") OR ("spinal opioids") OR ("spinal morphine") OR ("neuraxial analgesia") OR ("intrathecal analgesia") OR ("spinal analgesia") OR ("neuraxial morphine") OR ("neuraxial opioids")) AND (("postoperative pain") OR ("perioperative pain") OR ("respiratory depression") OR ((perioperative OR postoperative) monitoring) OR ("dose response") OR (guidelines) OR (safety)). Results were filtered to include articles available in English and published within the last 20 years.

**Figure 1:**
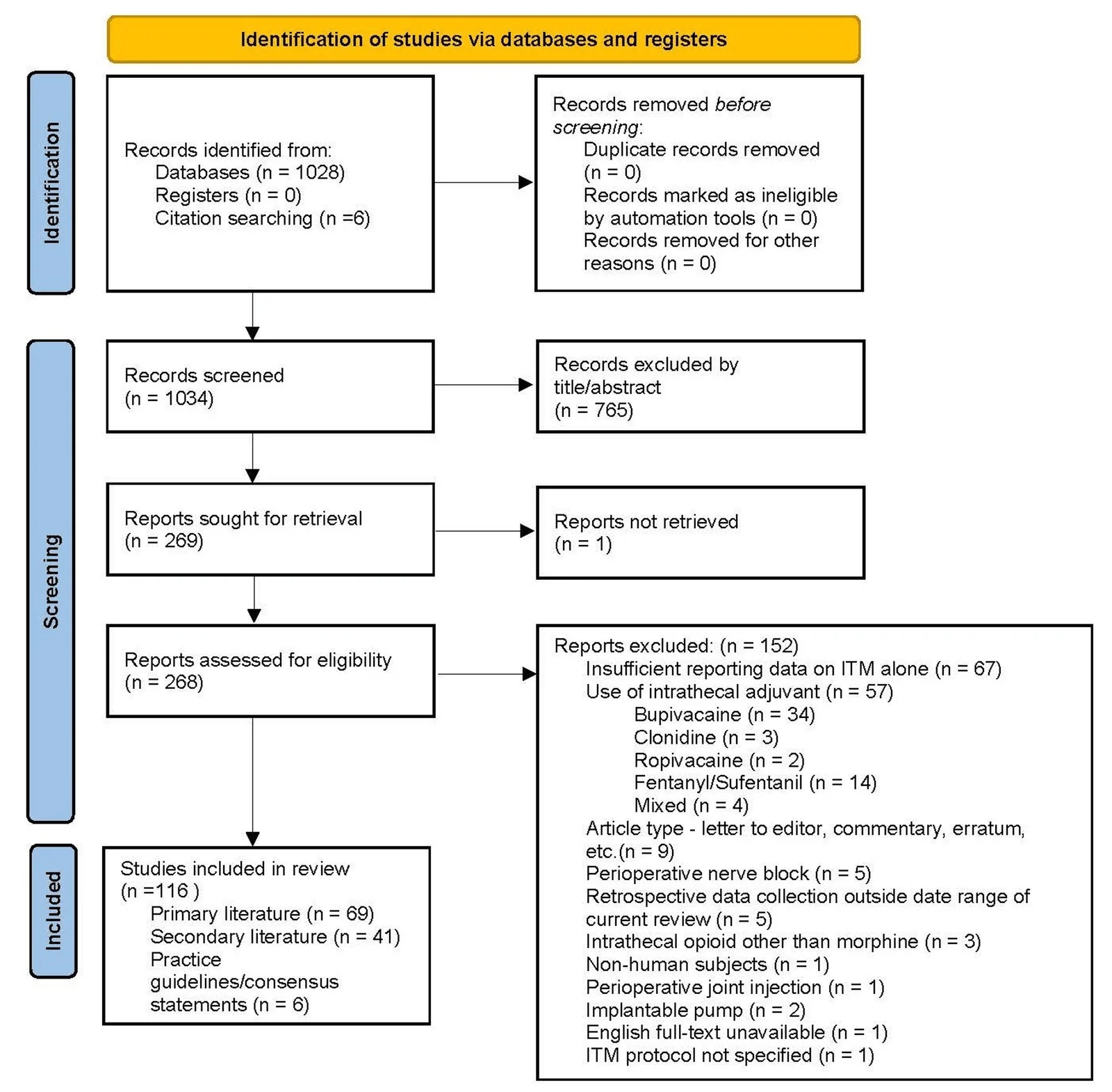
Structured literature search and article selection process n denotes the number of studies.

We included clinical trials, cohort studies, case series, case reports, systematic reviews, meta-analyses, narrative reviews, and clinical practice guidelines reporting on the use of ITM in surgical settings available in English and dated within the last 20 years. We included studies evaluating the efficacy of ITM, studies comparing ITM with systemic or epidural opioids, and studies evaluating the pharmacological properties of ITM. We excluded studies focused on implantable pumps, animal studies - unless directly reporting on clinical pharmacology - and studies involving intrathecal adjuvants or regional techniques. Articles were first reviewed by title and abstract for eligibility. The full texts of potentially eligible articles were then reviewed. Following the initial search and screening process, 1028 hits were returned, and 116 were included (Figure 1).

Two authors independently conducted the selection of relevant studies based on the objective criteria defined in the methods. To reduce the risk of bias, we clearly defined inclusion and exclusion criteria prior to the literature search. No formal risk-of-bias assessment was performed.

Pharmacology of ITM

Opioids have complex kinetics in the intrathecal space and follow a multicompartmental pattern of distribution [23]. They travel along a caudal-cephalic gradient in the CSF, undergo spinal diffusion to bind to specific opioid receptors in grey matter and nonspecific receptors in white matter, and diffuse toward the epidural space, where they bind to lipophilic structures. The clinical characteristics of each opioid are determined by the sum of these actions.

**Figure 2:**
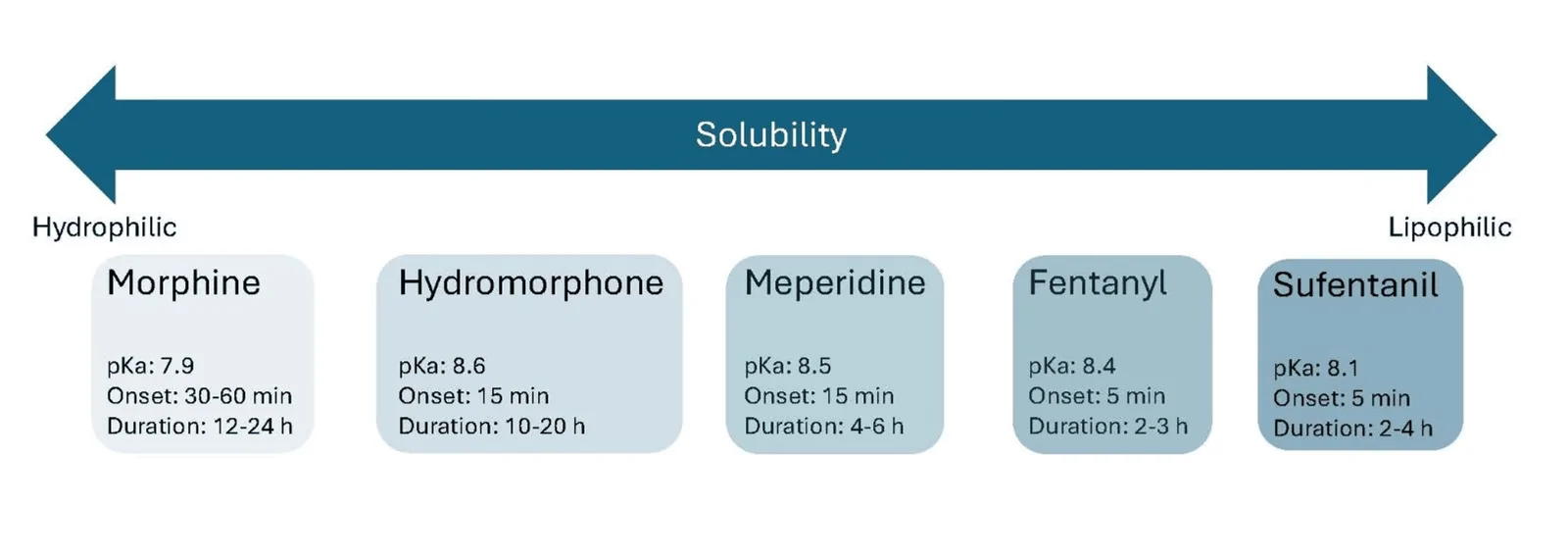
Ionization, solubility, onset and duration of intrathecal opioids Hydrophilic opioids (i.e., morphine) have a slower onset and prolonged duration of action. Lipophilic opioids (i.e., fentanyl) demonstrate rapid onset and shorter analgesic duration. Source: Ionization [27-29], Solubility [28,30], Onset and duration [31].

Morphine has an intrathecal-to-intravenous potency ratio ranging from 1:100 [21,24] to 1:300 [17,23]. Morphine is a weak base with a pKa of 7.9 and a 76% ionized fraction at a physiological pH of 7.4 [14]. Its two hydroxyl functional groups are able to participate in hydrogen bonding with water, rendering morphine a hydrophilic molecule [25]. While lipophilic drugs are rapidly redistributed from the intrathecal compartment toward surrounding lipophilic spaces, morphine remains at a sustained high concentration in the CSF, leading to a prolonged clinical effect and a risk of delayed respiratory depression due to increased cephalad spread reaching the brainstem (Figure 2) [15,23,26].

There is currently no standardized approach to ITM dosing. While doses up to 4000 µg have been described, doses used in contemporary practice rarely exceed 1000 µg in a single administration. Dose categories based on the available literature have been described as ultra-low (< 50 µg or < 1.5 µg/kg), low (50 µg to < 150 µg or 1.5 µg/kg to < 5 µg/kg), medium (150 µg to < 500 µg or 5 µg/kg to < 7 µg/kg), and high (≥ 500 µg or ≥ 7 µg/kg) [13,32,33]. Most modern studies employ doses ranging from 100 to 500 µg, with doses between 75 and 150 µg often cited as providing an optimal balance between analgesic efficacy and adverse effects [7,17]. ITM is administered in the lumbar region to minimize the risk of spinal cord injury. As a result, dosing requirements may vary depending on the surgical procedure, with surgeries involving more cephalad dermatomes, such as cardiothoracic surgery, often employing higher ITM doses than procedures involving lumbar or lower abdominal regions to achieve effective analgesia [8,15].

Clinical benefits

ITM has been shown to attenuate the physiological stress response to pain due to reduced activation of the sympathetic nervous system [34-36]. Karaman et al. observed significantly lower plasma epinephrine, norepinephrine, and glucose levels in patients receiving ITM compared with patient-controlled analgesia (PCA) for total abdominal hysterectomy [34]. Roediger et al. also observed lower plasma catecholamine concentrations in patients receiving ITM for coronary artery bypass compared with PCA alone [35]. Similarly, a trial by Salam Omara and Amer found lower plasma cortisol levels 30 minutes after incision in laminectomy patients who received ITM preoperatively [36].

Analgesia provided by ITM is estimated to last between 18 and 48 hours, delaying the need for postoperative rescue analgesia [23,33,37]. Both meta-analyses and randomized trials consistently demonstrate prolonged time to first rescue analgesic compared with IV opioids, with delays ranging from hours to more than two postoperative days depending on surgical procedure [38-41]. A 2024 meta-analysis reported a mean increase in time to first postoperative analgesic request of 9.62 hours (95% CI 8.13, 11.11) in patients receiving ITM versus those receiving systemic opioids, a finding consistent with several other similar analyses conducted in the preceding five years [42]. There are studies in which ITM recipients required little to no rescue analgesia during the first postoperative day while reporting lower pain scores [43]. In addition to improving pain control, ITM substantially reduces cumulative postoperative opioid consumption compared with systemic administration, with several studies reporting 50%-75% reductions in opioid utilization across diverse surgical populations [37,44-51]. Recent meta-analyses of randomized controlled trials have quantified this reduction, with a 2023 article reporting a mean difference in morphine equivalents of -20.13 (95% CI -30.74, -9.52) at 24 hours postoperatively [32,42,46,52-54].

Patients receiving ITM often report lower levels of postoperative pain on psychometric measurement tools than those receiving IV morphine. This effect has been documented as soon as the first postprocedural hour and up to 72 hours [34,35,39-41,43,52,55-68]. Additionally, there is evidence that the prolonged analgesic effect of ITM enhances postoperative recovery and shortens length of hospital stay. De Bie et al. demonstrated that ITM improved time to first mobilization by as much as 16.8 hours (p = 0.002) for patients undergoing lumbar fusion [57]. Additionally, Hong et al. observed that time to Foley catheter removal was reduced by as much as 19 hours (p < 0.001) for adolescents undergoing posterior spinal fusion [61]. A retrospective review of 373 adolescents undergoing lumbar fusion observed significantly earlier return to ambulation and oral intake, Foley catheter removal, and time to first bowel movement in patients receiving ITM compared with those on PCA [51]. ITM also has the potential to shorten overall length of hospital stay. While not universally observed, use of ITM has facilitated postoperative discharge anywhere from 1 day [57,69] to 3.44 days earlier than systemic opioids [51,55,57,61,67,69,70].

Analgesic efficacy by surgical population

Because ITM is administered in the lumbar region to mitigate the risk of spinal cord injury, the distance between the injection site and the surgical dermatome varies substantially across procedures. In the absence of standardized dosing recommendations, this anatomic constraint has contributed to procedure-specific dosing practices and heterogeneity in ITM efficacy and adverse effects. The following section summarizes available evidence by surgical category to contextualize the observed differences in dosing and analgesic benefit. 

Cardiothoracic

Recent literature has predominantly reported on the use of high-dose ITM (≥ 500 µg or 7 µg/kg) for cardiac procedures, which performs superiorly to systemic opioids in measures of postoperative pain intensity, cumulative opioid consumption, duration of analgesia, and length of stay in the ICU and hospital [35,38,69,71,72]. However, low-to-medium dose ITM (< 500 µg or < 7 µg/kg) has also demonstrated efficacy in improving markers of pain management [32,59,63,73,74]. Only one study utilized ultra-low-dose ITM at 1.5 µg/kg; this was for minimally invasive valve repair and was administered at a higher lumbar level (L1-3) than is typical [63].

A 2025 scoping review asserted that ITM effectively reduces pain for 24 hours for thoracic surgery, with an opioid-sparing effect up to 48 hours [75]. Effective analgesia for minimally invasive procedures has been noted with doses as low as 200 µg [68], while dosing for open-chest procedures ranged up to 1000 µg [76]. A 2025 dose comparison study on video-assisted thoracic surgery found that 10 µg/kg was more effective at reducing overall PCA morphine consumption and pain scores at 18 and 24 hours than 7 µg/kg without an increase in side effects [77]. In addition, 10 µg/kg has been documented to preserve peak expiratory flow rates after thoracotomy compared with control [44].

Abdominal

The clinical efficacy of ITM has been documented for a wide array of abdominal procedures. In studies of abdominal surgery, 100 µg to 300 µg doses have demonstrated efficacy over PCA in colorectal surgery, gastrectomy, and laparotomy [45,78,79]. A study comparing 200 µg, 500 µg, and 1000 µg doses for abdominal cancer surgery observed superior analgesic effects in the higher dosing groups for up to 48 hours, though these patients also experienced higher incidences of adverse effects [80].

Orthopedic

Minimal literature exists on the use of ITM in isolation for orthopedic surgeries; it is often administered in solution with a local anesthetic. Additionally, no studies exist on the use of ITM for procedures of the upper extremity. Several meta-analyses have demonstrated the clinical efficacy of low-dose ITM in postoperative analgesia for total joint arthroplasties of the lower extremity; a 2021 meta-analysis established the existence of an analgesic ceiling effect beyond 100 µg ITM [54,81,82]. This dose provided the optimal balance of analgesia and side effects, as 100 µg ITM was also found to be the threshold dose for PONV. The ability to provide effective analgesia at such low doses may be related to the proximity of intrathecal injection to the spinal levels of the lumbar and sacral plexuses that innervate the lower extremity.

The 2008 procedure-specific postoperative pain management (PROSPECT) guidelines for total knee arthroplasty recommended the use of ITM with local anesthetic, though not as the first choice of analgesia due to greater potential for adverse events compared with femoral nerve block [83]. The 2021 PROSPECT guidelines for THA could not reach unanimous consensus regarding the use of 100 µg ITM and cautioned clinicians to weigh the benefits and risks of pruritus and PONV associated with its use [84].

Spinal

ITM has demonstrated efficacy in treating postoperative pain after spinal surgery in both adults [24,85] and children [17,79]. Doses ranging from 100 µg to 400 µg have been utilized in lumbar fusion surgeries [41,86]; for posterior spinal fusion, ITM at doses from 1.5 µg/kg to 6 µg/kg was superior to systemic opioids, with 6 µg/kg leading to a reduction in hospital length of stay by as much as 1.8 days [51,70]. ITM has not demonstrated superiority over systemic opioids for osteotomy or rhizotomy [87,88]. Several meta-analyses have suggested that use of ITM for spinal procedures comes with significant benefit and no increase in adverse effects [53,89-91].

Urological

ITM appears to provide superior pain control to PCA or control at doses from 200 µg to 400 µg following kidney transplant or nephrectomy [40,62,92-94]. There may be a protective effect against developing eGFR < 60 in kidney transplant donors who receive 200 µg of ITM prior to surgery [95] and against delirium in kidney transplant recipients when the dose range is extended up to 400 µg [94].

Use of ITM has also been explored for open or robot-assisted prostatectomy. For radical retropubic prostatectomy, 200 µg was found to be effective without increasing the incidence of adverse effects. In fact, patients who received ITM had a 61% reduction in the incidence of nausea compared with controls [65]. A dose of 250 µg showed similar efficacy with no increase in adverse effects [93]. Administration of 300 µg ITM prior to robot-assisted laparoscopic prostatectomy was found to provide effective analgesia without adverse complications [43].

Obstetric and Gynecological

ITM has a well-established role in obstetric practice and is part of standard practice in post-cesarean delivery analgesia [10,96]. The 2020 PROSPECT guidelines for elective cesarean section currently recommend preoperative administration of 50 µg to 100 µg ITM as part of a multimodal pain regimen [97]. Doses of ITM < 100 µg are thought to provide optimal analgesia without increasing the risk of adverse effects, including respiratory depression or urinary retention [98-100].

ITM has also demonstrated the ability to expedite recovery in gynecological oncological surgery [48,101]. A 2023 article found that 125 µg provided superior analgesia to IV opioids for laparoscopic gynecological surgery [60]. A dose of 5 µg/kg has also demonstrated efficacy in the context of total abdominal hysterectomy but increased the incidence of postoperative nausea, vomiting, and pruritus [34].

Ambulatory Surgery

ITM is not currently considered to be an appropriate analgesic choice for ambulatory surgery due to its slow onset, long duration of analgesia, and potential for delayed-onset respiratory depression [14]. Urinary retention is an additional consideration. In one study using ITM doses of 15 µg to 250 µg, 20%-40% of subjects experienced urinary retention two hours after administration of ITM, though rates improved to less than 10% 24 hours after injection [102].

Adverse effects

Respiratory Depression

High concentrations of mu opioid receptors in the ventral medulla are important in physiological regulation of respiration; they are also involved in opioid-induced respiratory depression [15]. Direct application of opioids to chemosensitive centers of the medulla can induce significant respiratory depression. Recent studies have identified that opioid activity in the pre-Bötzinger complex causes hyperpolarization of neurons expressing neurokinin-1 receptors. This disrupts normal respiratory rhythm generation and decreases respiratory rate. ITM-associated respiratory depression is thought to be bimodal, with early respiratory depression occurring within two hours of administration and delayed respiratory depression occurring approximately 6-12 hours after administration [100,103-105].

**Figure 3:**
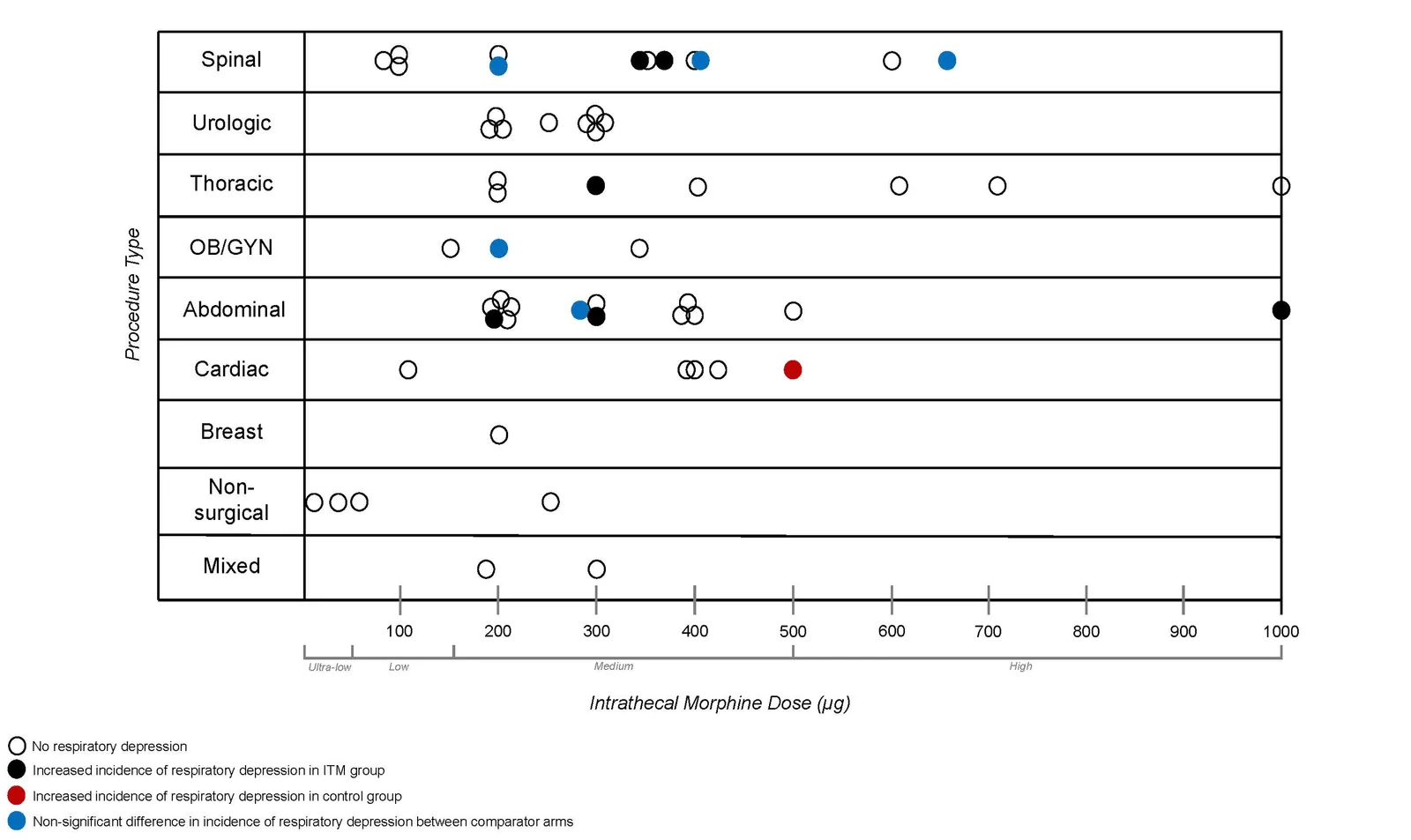
Incidence of respiratory depression associated with intrathecal morphine across surgical types Each point represents reported intrathecal morphine (ITM) doses by procedure type. Studies are displayed with equal visual weight regardless of sample size and do not account for differences in study design, monitoring methods, or respiratory depression severity. Detailed definitions of respiratory depression, sample sizes, and the number of patients with respiratory depression are provided in Appendix 1.

Large retrospective and prospective data analyses indicate the incidence of respiratory depression following receipt of 150 µg-800 µg ITM to range from 0.26% to 3%, which is comparable to the risk associated with systemic opioids [15,106]. ITM-induced respiratory depression is suggested to be dose-dependent (Figure 3), with low- to intermediate-dose ITM administered at the lumbar level largely metabolized or redistributed before reaching the brainstem in concentrations sufficient to cause respiratory depression [75]. In a 2024 meta-analysis, ITM administered at doses < 7 µg/kg had an incidence of respiratory depression similar to that associated with systemic opioids (0.61%, n = 164 vs. 0.52%, n = 387, respectively), while ITM > 19 µg/kg (n = 46) was associated with a 15.2% incidence of respiratory depression [42]. This was consistent with a 2020 meta-analysis that observed most cases of respiratory depression to have occurred in the context of ITM administered in 12 µg/kg and 50 µg/kg doses [104]. It is important to note, however, that doses within this range have been used without observing respiratory depression [76].

Appendix 1 lists all the trials that investigated respiratory depression stratified by surgical type and ITM dose concentration. Of 47 studies, 35 reported no respiratory depression [34,35,37-41,43,44,49-51,55,57-59,61-66,68,73,76,77,80,85-87,92,93,95,101,102,107-116]. Seven studies reported respiratory depression; however, the incidence was low (five patients or fewer) [35,40,45,80,85,112,115]. The study reporting the highest percentage of patients with respiratory depression observed an incidence of 26.7% after receiving 500 µg ITM; however, the incidence of respiratory depression in the control group was 80% [35]. Five studies reported no significant difference in the incidence of respiratory depression between patients receiving ITM and those receiving either epidural opioids, IV opioids, or sham procedures [58,61,101,107,110].

Recent evidence suggests that ITM may preserve respiratory function when compared with IV administration. Askar et al. observed better preservation of peak expiratory flow rates in patients who received 10 µg/kg ITM for thoracotomy compared with patients randomized to receive IV PCA [44]. Roediger et al. observed the same effect in patients who received 500 µg ITM for coronary artery bypass graft surgery (CABG); these patients also had higher postoperative PaO_2_/RiO_2_ ratios [35]. Administration of 5 µg/kg ITM has also demonstrated a protective effect on forced expiratory volume in one second and forced vital capacity in patients undergoing CABG or single valve replacement surgery [74].

Understanding the true incidence of respiratory depression following ITM exposure is difficult in part due to the lack of a formal definition. As such, parameters used to detect respiratory depression vary greatly. Of the studies reporting on respiratory depression included in this review, there was notable heterogeneity among reporting measures. Definitions included bradypnea ranging from 6 breaths/min [97] to <14 breaths/min [113], hypercarbia [59], hypoxemia ranging from SpO_2_ 88% [107] to SpO_2_ <95% [28,34], Ramsay sedation score [77], and naloxone administration [76,108]. Development of a standard definition for respiratory depression would be an important step in uncovering the true incidence of ITM-induced outcomes.

Pruritus

Pruritus is one of the most common adverse effects seen with ITM use [17]. Neuraxial morphine-induced itch was once assumed to be mediated by peripheral histamine release. Animal studies have disproven this theory and instead implicated central disinhibition of Oprm1-expressing inhibitory neurons in the pathogenesis [117]. GABAergic neuronal disinhibition permits excitatory neurons expressing gastrin-releasing peptide receptor to trigger itch [118].

Pruritus is thought to be dose-dependent, with the greatest risk occurring at doses >100 µg ITM [104,119-121]. A 2021 meta-analysis reported an incidence of 38.8%, while other studies have reported incidences ranging from 0% to 100% [17]. Despite this, most cases do not require treatment, and severe pruritus is only thought to occur in 1% of patients. When treatment is required, butorphanol or low-dose naloxone can be administered to reverse symptoms without a decrease in analgesic effect [90,122,123]. Antihistamines have historically been used for treatment with some clinical efficacy; however, their effect appears to reduce itch secondary to somnolent effects [117,123].

Nausea/Vomiting

Postoperative nausea and vomiting (PONV) are common adverse effects associated with opioids. ITM is thought to cause PONV through activity at the chemoreceptor trigger zone in the area postrema, where stimulation increases vestibular sensitivity and delays gastric emptying [118].

Previous meta-analyses have identified 100 µg to be the threshold ITM dose for PONV [82,100]. Further literature is mixed on whether PONV displays a dose-dependent relationship, and a 2024 meta-analysis suggests that there may be an all-or-nothing mechanism underlying symptoms [118]. A 2013 meta-analysis observed a higher incidence of vomiting with doses of ITM < 300 µg (RR = 3.1, 95% CI 1.5, 6.4) compared with studies of ITM ≥ 300 µg (RR = 1.3, 95% CI 0.9, 1.9) [106]. A 2013 clinical trial involving administration of 200 µg ITM for radical retropubic prostatectomy observed lower rates of PONV in patients receiving ITM (3.7%) than in control patients (64.7%) (p = 0.001) [65].

Sedation

According to a 2025 meta-analysis, over-sedation is rare with doses of ITM < 150 µg and is not increased with any degree of clinical significance in doses < 500 µg [18]. This finding is consistently represented in high-level evidence published within the last two decades [67,104]. Advanced sedation generally precedes respiratory depression, and thus the two adverse effects should be considered in parallel [103,105].

Urinary Retention

Intrathecal opioids are known to impair bladder function by interfering with detrusor muscle contraction [118]. This effect is believed to be related to spinal cord inhibition of acetylcholine release, which typically enhances detrusor muscle contractility and urge sensation [120]. When urinary retention does occur, it is likely to resolve spontaneously within 10-20 hours. While this is reassuring, the inability to micturate spontaneously is considered to be one of the most distressing non-respiratory complications for patients receiving ITM, and it is important to monitor bladder function, clinically or with ultrasound, to prevent overdistension and reduce the risk of neurogenic bladder [17].

A 100 µg ITM has been suggested as the threshold dose for urinary retention, and the phenomenon does not appear if patients receive a urinary catheter for the first 24 hours after injection [75].

Additional Considerations

ITM is contraindicated in patients with severe thrombocytopenia and coagulopathy due to the risk of spinal epidural hematoma [124]. Uncorrected hypovolemia and elevated intracranial pressure are additional contraindications [125].

Practice guidelines for dose-stratified monitoring

Guidelines

The American Society of Anesthesiologists (ASA) published the first set of practice guidelines addressing respiratory depression associated with neuraxial opioids in 2009 [126], which were revised in 2016 [16]. The ASA recommends monitoring respiratory rate, depth, oxygenation, and level of consciousness at least once per hour for the first 12 postoperative hours and once every two hours for the following 12 hours. After 24 hours, monitoring frequency should be determined by clinical status.

The Society for Obstetric Anesthesiology and Perinatology (SOAP) released an alternative risk-stratified set of guidelines in 2019. These guidelines recommend the following: (a) no additional monitoring beyond routine institutional policies for ITM doses < 50 µg, (b) respiratory and sedation assessments every 2 hours for 12 hours postoperatively for ITM doses > 50 µg to ≤ 150 µg, and (c) adherence to ASA guidelines for ITM doses > 150 µg [13]. In patients with comorbidities or perioperative risk factors, SOAP advises that the frequency, duration, and modality of respiratory monitoring be guided by clinical judgment, institutional policy, or the ASA guidelines.

Literature Findings

According to the ASA, there remains insufficient evidence to define optimal monitoring intervals or thresholds for detecting respiratory depression [16]. Conversely, SOAP concluded that respiratory depression is exceedingly rare beyond 12 hours following administration at contemporary ITM doses [13,45,110,115].

Across studies, respiratory depression was an uncommon event, typically occurring in patients with advanced age or significant comorbidities rather than being dose-dependent. In a retrospective study of 484 patients, nearly all adverse respiratory events occurred in older individuals with at least one comorbidity, while ITM dose varied widely and did not appear to be associated with the event [111]. Similarly, a dose of 1000 µg ITM resulted in only one case of respiratory depression in a frail ASA III patient with ovarian cancer [80]. As summarized in Appendix 1, the incidence of respiratory depression was low in patients receiving doses of ITM 300 µg or lower. Respiratory depression was also seen in patients not receiving ITM.

Case reports further emphasize that respiratory compromise often results from multifactorial causes rather than ITM dose alone. An example of this can be seen in a case study of a 46-year-old male with intractable pain who received 300 µg ITM alongside epidural PCA morphine and developed oversedation and aspiration pneumonitis despite a preserved respiratory rate [105]. Similarly, Suksompong et al. reported a 69-year-old female who developed hypercapnia and required reintubation after receiving 300 µg ITM despite a preserved respiratory rate [115].

These data suggest that the incidence and timing of respiratory depression after low-to-moderate doses of ITM are comparable to those observed with IV opioids. Monitoring standards requiring hourly assessments for up to 24 hours may be disproportionate to the observed risks at contemporary dosing levels. Evidence supports a more individualized, risk-stratified approach, consistent with SOAP recommendations. This is particularly important for resource-limited settings where conservative monitoring protocols may discourage the safe and effective use of ITM.

Considerations for special populations

Populations at increased risk of adverse effects from neuraxial opioid administration include the elderly, patients with OSA, those receiving supplemental oxygenation, and individuals with severe obesity [19]. Advancing age is associated with reduced hepatic metabolism, renal blood flow, and glomerular filtration rate [127]. Age-related increases in adipose tissue also expand the volume of distribution and prolong elimination time for lipophilic opioids [17]. Consequently, these drugs may exhibit greater potency and longer duration of action in older patients.

Special consideration should be given to patients with baseline respiratory dysfunction. Obesity increases the mechanical load on the respiratory system, reducing tidal volume and blunting the chemoreflex response to carbon dioxide [128]. The risk of paradoxical carbon dioxide narcosis may be heightened when these effects are combined with opioid-induced respiratory depression [129,130]. OSA is characterized by relaxation of the soft palate and intermittent upper airway obstruction. Opioid-induced depression of airway muscle tone may exacerbate these episodes [130].

Limitations

An important limitation of our study is the exclusion of trials using adjuvant intrathecal anesthetics, which limits our generalizability. We did not perform a multi-database search or a formal risk-of-bias assessment, as would be done in a systematic review; these represent important limitations. Another limitation includes the heterogeneity in perioperative pain management among non-ITM controls across studies. However, a study’s intervention and control groups were managed under the same strategy, which strengthens internal validity. There was also heterogeneity in definitions of respiratory depression, sample sizes, and monitoring protocols for the detection of respiratory depression. This limitation will persist until a standardized approach is developed that defines respiratory depression, emphasizing the importance of future research in this area.

## Conclusions

ITM remains a reliable and effective component of multimodal analgesia, consistently shown to reduce postoperative systemic opioid requirements. At contemporary doses below 300 µg, the incidence of respiratory depression appears low and comparable to that reported with IV PCA. When respiratory events occur, they most commonly arise within the first 12 hours after administration and are strongly associated with identifiable patient-level risk factors, including advanced age, obesity, and OSA. Despite substantial evidence supporting the safety of ITM at lower doses, monitoring practices and dosing strategies remain highly variable across institutions. A more standardized, risk-stratified approach that tailors monitoring to ITM dose and patient-specific factors may improve broader utilization.

Further progress in optimizing safe and effective use of ITM will depend on establishing uniform definitions for respiratory depression and evaluating practice-level data across diverse institutions and health systems, including those in resource-limited settings. Understanding how ITM is universally implemented will help clarify barriers to adoption, inform refinement of dosing and monitoring practices, and support the development of consistent, evidence-based standards for postoperative pain management.

## Appendices

Appendix 1

**Table 1:** Incidence of respiratory depression associated with intrathecal morphine across surgical types Abbreviations: ND: non-significant difference in incidence of respiratory depression between comparator arms (non-significant p-value); RR: respiratory rate; ITM: intrathecal morphine; SpO_2_: peripheral oxygen saturation; SaO_2_: arterial oxygen saturation; EREM: extended-release epidural morphine; PaCO_2_: arterial carbon dioxide tension; ASA: American Society of Anesthesiologists

Surgical Type	Author	Study Type	# of Patients (n)	ITM Dose	Analgesic Outcomes	Incidence of Respiratory Depression	Respiratory Depression Definition
Spinal							
	Araimo Morselli et al. [55]	Randomized controlled trial	50	100 µg	ITM was associated with lower NRS scores, earlier mobilization, and shorter hospitalization than IV morphine.	None	SpO_2_ and arterial blood gas analysis
	Audlin et al. [87]	Retrospective review	17	600 µg	No statistical difference in postoperative pain or side effects noted between ITM, IV opioids, or oral opioids.	None	-
	De Bie et al. [57]	Retrospective cohort study	60	100 µg	ITM was associated with lower cumulative morphine consumption and VAS scores with earlier mobilization and earlier discharge compared to control.	None	-
	Sarwahi et al. [51]	Retrospective review	373	1.5 µg/kg (avg 82.65 µg)	ITM was associated with shorter length of stay, earlier ambulation, and lower VAS scores compared to control.	None	-
	Wang et al. [41]	Randomized controlled trial	92	200 µg	ITM was associated with lower VAS scores, PCA use, and supplemental analgesic demands with no increase in adverse effects compared to control.	None	-
	Yen et al. [116]	Randomized controlled trial	32	3.5 µg/kg (350 µg max)	ITM resulted in lower PCA use with no difference in pain scores compared to control.	None	RR < 9 breaths/min
	Ziegeler et al. [86]	Randomized controlled trial	46	400 µg	ITM resulted in improved VAS scores at rest 4 and 8 hours after surgery with significantly lower cumulative PCA requirements compared to control.	None	SaO_2_ and blood gas analysis
	Ibach et al. [112]	Retrospective, cross-sectional, observational study	44	average 369 µg	Patients experienced improved analgesia for at least 12 hours following ITM administration.	11.4% (5 patients)	RR < 10 breaths/min
	Poblete et al. [85]	Prospective proof-of-concept study	20	7 µg/kg (140-550 µg)	ITM for postoperative analgesia after resection of cervical and thoracic spinal cord tumors was observed to be safe and effective.	5% (1 patient). Respiratory depression subsided quickly and without consequence or further intervention.	RR < 8 breaths/min
	Cohen et al. [107]	Randomized controlled trial	71	7.5 µg/kg (avg 402 µg)	No difference in total morphine consumption or time until first PCA demand between EREM and ITM. EREM provided longer duration of analgesia and was associated with less opioid induced pruritus.	ND in incidence between ITM vs extended-release epidural morphine (EREM). 24% (17 patients) of ITM pts, 15% (11 patients) of EREM pts.	SpO_2 _< 88%
	Dhaliwal et al. [58]	Randomized controlled trial	150	200 µg	ITM was associated with reduced postoperative opioid requirements and VAS scores compared to control.	ND in incidence between ITM vs sham saline injection. No significant differences in respiratory rates or oxygen saturation between treatment groups in the 24hrs postop.	RR < 10 breaths/min
	Hong et al. [61]	Retrospective review	40	12 µg/kg (avg 667.2 µg)	Use of ITM in addition to routine administration of nonopioid analgesics facilitated early transition to oral analgesics, early ambulation, and early discharge compared to epidural hydromorphone.	ND in incidence between ITM vs epidural hydromorphone. 33% of patients in both groups required nasal oxygen cannulation at some point after PACU discharge, however, none required naloxone or additional treatment for hypoxemia.	SpO_2_ < 92%
Urologic							
	Swisher et al. [66]	Retrospective case-control	27	300 µg	ITM was associated with decreased opioid consumption and NRS scores compared to control.	None	-
	Park et al. [95]	Retrospective review	366	200 µg	ITM was associated with better functional recovery of remnant kidney in living donors with favorable analgesic results on NRS.	None	-
	Nuri Deniz et al. [65]	Randomized controlled trial	56	200 µg	ITM provided significant reduction in postoperative VAS scores, rescue analgesia, and postoperative nausea without serious adverse effects compared to control.	None	RR < 8 breaths/min
	Kurzova et al. [93]	Randomized controlled trial	41	250 µg	ITM provided long-lasting analgesia and reduction in postoperative systemic administration of opioids compared to control. Adverse effects were comparable between groups.	None	Bradypnea or SpO_2_ < 90%
	Kim et al. [92]	Retrospective review	272	200 µg	ITM was associated with lower NRS scores and cumulative opioid consumption compared to control.	None	-
	Kim et al. [62]	Randomized controlled trial	45	300 µg	ITM was associated with lower postoperative pain on NPS with significantly lower cumulative morphine consumption compared to control.	None	At least one of the following variables: respiratory rate < 8 breaths/min, ­SpO_2_ < 90% or PaCO_2_ > 70 mmHg
	Bae et al. [43]	Randomized controlled trial	30	300 µg	ITM was associated with lower NPS scores and cumulative morphine consumption compared to control.	None	At least one of the following variables: respiratory rate < 8 breaths/min, SpO_2_ < 90% or PaCO_2_ > 70 mmHg
	Boonmak et al. [56]	Randomized controlled trial	80	300 µg	ITM was associated with lower cumulative morphine consumption and NRS scores with no significant difference in patient satisfaction with pain management compared to control.	None	-
Thoracic							
	Askar et al. [44]	Randomized controlled trial	33	10 µg/kg (avg 712.4 µg)	ITM was associated with lower VAS scores and morphine consumption compared to control with better preservation of respiratory function.	None	-
	Okbaz et al. [77]	Randomized controlled trial	46	7 µg/kg (avg 429.1µg)	ITM at 10µg/kg was associated with lower postoperative morphine consumption and postoperative pain scores at 18 and 24 hours compared to ITM at 7µg/kg.	None	SpO_2_ < 90 on room air, RR <10 breaths/min, or Ramsay sedation scale score >3
				10 µg/kg (avg 613 µg)			
	Vijitpavan et al. [68]	Randomized controlled trial	38	200 µg	ITM was associated with lower NRS scores and cumulative dose of postoperative morphine compared to control.	None	RR < 8 breaths/min
	Ward et al. [76]	Retrospective review	84	Initial injection 1000 µg, 2560 µg over 48hrs	ITM was found to be an efficacious and safe analgesic method in post-thoracotomy patients with no serious complications or sequelae at 6-month follow up.	None	RR < 8 breaths/min and/or requiring naloxone
	Suksompong et al. [115]	Randomized controlled trial	40	200 µg	No statistical difference was found in 24-hours postoperative meperidine usage between groups.	5% (1 patient) in 300 µg group. Pt developed respiratory acidosis with RR 10 breaths/min 44 minutes after extubation.	One patient in 300 µg group developed respiratory acidosis with RR 10 breaths/min 44 minutes after extubation
				300 µg			
Obstetric & Gynecological							
	Crowgey et al. [108]	Retrospective review	5336	50-250 µg (avg 150 µg)	The upper 95% confidence limit for respirator depression was found to be 0.07%.	None	Naloxone administration for reversal of RD
	Karaman et al. [34]	Randomized controlled trial	24	5 µg/kg (avg 344 µg)	ITM was associated with lower VAS scores, total morphine consumption, and systemic stress response compared to control.	None	RR < 10 breaths/min
	Bang et al. [101]	Randomized controlled trial	68	200 µg	ITM was associated with lower cumulative opioid consumption and higher patient satisfaction regarding pain control compared to control.	ND in incidence between ITM and sham group – sham procedure involved 2 minutes of applied pressure with a capped needle without dural puncture. 6.1% (2 patients) of ITM group. 3% (1 patient) of sham group.	RR < 8 breaths/min or need for supplemental oxygen to maintain SpO_2_ 92%
Abdominal							
	Lee, S. et al. [114]	Prospective randomized non-inferiority trial	55	300 µg	ITM 300 µg exhibited non-inferior postoperative analgesia with lower incidence of PONV compared to 400 µg.	None	RR < 8 breaths/min or SpO_2_ < 90%
				400 µg			
	Niewiński et al. [64]	Randomized controlled trial	36	400 µg	No differences in NRS scores, pain severity, cumulative opioid consumption, or early recovery was observed between ITM and IV morphine.	None	-
	Blay et al. [37]	Randomized controlled trial	32	200 µg	ITM was associated with decreased intraoperative sufentanil, cumulative morphine consumption and VAS scores compared to control.	None	-
	De Pietri et al. [109]	Retrospective case control	50	200 µg	ITM was associated with lower cumulative morphine consumption and VAS scores, earlier mobilization, and earlier discharge compared to control.	None	Hypercapnia or hypoxia
	Ko et al. [39]	Randomized controlled trial	40	400 µg	ITM was associated with lower VAS scores and cumulative opioid consumption compared to control.	None	RR < 8 breaths/min
	Kuppusamy et al. [50]	Randomized controlled trial	60	200 µg	ITM was associated with lower cumulative morphine consumption compared to control. There was no statistically significant difference in VAS scores.	None	RR < 8 breaths/min or SpO_2_ < 90%
	Beaussier et al. [45]	Randomized controlled trial	52	300 µg	ITM was associated with decreased pain intensity and daily IV morphine consumption compared to control.	7.7% (4 patients). 1 pt experienced deep sedation with respiratory depression and inhalation of gastric contents.	RR < 10 breaths/min
	Fares et al. [80]	Randomized controlled trial	90	200 µg	Time to first analgesic request was prolonged with 500 µg and 1000 µg compared to 200 µg. Mean tramadol consumption was lower with 500 µg and 1000 µg compared to 200 µg. VAS scores were lower with 1000 µg compared to 500 µg and 200 µg.	3.3% (1 patient) in 1000 µg group. Pt was 65 years old, frail, and ASA III.	RR < 10 breaths/min
				500 µg			
				1000 µg			
	Mittal et al. [40]	Randomized controlled trial	60	200 µg	ITM was associated with lower NRS scores, cumulative fentanyl consumption, and lower total rescue analgesia compared to control.	13.3% (4 patients) in ITM group, 3.7% (1 patient) in control group.	SpO_2_ < 92%
	Dichtwald et al. [110]	Randomized controlled trial	49	4 µg/kg (avg 288 µg)	ITM was associated with lower NRS scores and reduced need for rescue analgesia compared to IV opioids.	ND in incidence between ITM vs IV remifentanil. 3% of all patients had non-life-threatening respiratory depression.	RR < 10 breaths/min and/or PaCO_2_ > 60 mmHg
Abdominal, Urologic, or Thoracic							
	González-Santos et al. [111]	Retrospective review	484	180 µg average (400 µg max)	51.03% of patients required postoperative rescue analgesia with IV morphine with an average dose of 6.98 mg, this was significantly higher in thoracic surgery patients compared to general surgery patients or urological surgery patients.	None	RR < 10 breaths/min, Sp0_2_ < 90%, or signs of drowsiness/sedation
Cardiac							
	Dhawan et al. [55]	Randomized controlled trial	79	5 µg/kg (avg 425 µg)	ITM was associated with lower cumulative morphine equivalents, VAS scores, and percent of time in severe pain compared to control.	None	Prolonged tracheal reintubation secondary to hypercarbia or escalation of respiratory support after extubation secondary to hypercarbia
	dos Santos et al. [73]	Randomized controlled trial	42	400 µg	ITM was associated with lower VAS scores, cumulative morphine consumption, and plasma morphine concentration compared to control.	None	-
	Elgendy et al. [38]	Randomized controlled trial	44	7 µg/kg (avg 392 µg)	ITM was associated with lower VAS scores and cumulative fentanyl consumption, and mean tramadol consumption compared to control. Time to first request for rescue analgesia was prolonged with ITM.	None	RR < 10 breaths/min
	Mukherjee et al. [63]	Randomized controlled trial	61	1.5 µg/kg (avg 118.5 µg)	ITM was associated with decreased demand for IV opioids and VAS scores compared to IV opioids.	None	PaCO_2_ (limit not defined)
	Roediger et al [35]	Randomized controlled trial	30	500 µg	ITM was associated with a 40% reduction in IV morphine and lower VAS scores at rest compared to control.	26.7% (4 patients) in ITM group, 80% (12 patients) in control group.	RR < 10 breaths/min
Breast							
	Karamese et al. [49]	Randomized controlled trial	62	200 µg	ITM was associated with lower cumulative morphine consumption than control; no different was noted in pain and satisfaction scores.	None	RR< 8 breaths/min and/or SpO_2_ < 90%
Non-surgical							
	Raffaeli et al. [102]	Randomized controlled trial	144	15 µg	Patients receiving ITM all experienced clinically significant pain relief on VAS scores with reduction in values ranging from 60% - 100%; only 25% of controls showed a reduction in VAS scores of over 50%.	None	RR < 6 breaths/min
				30 µg			
				60 µg			
				250 µg			
Pediatric							
	Keskin et al. [113]	Retrospective observational cohort study	100	5 µg/kg (300 µg max)	96% of patients did not require rescue analgesia, sleep interruption was not observed in 97% of cases, and nurses were satisfied or very satisfied with pain management in 99% of cases.	None	RR < 18 breaths/min in patients < 10 years old; RR < 14 breaths/min in patients > 10 years old; PaCO_2_ above 45 mmHg
